# Salivary Titanium Level as a Non-invasive Biomarker for Peri-Implantitis: A Prospective Study

**DOI:** 10.7759/cureus.97643

**Published:** 2025-11-24

**Authors:** Kavita Sharma, Swati Swati, Parween Jamil, Raghavendra Sumanth Phani Challa, Naveen Ambata, Rahul VC Tiwari, Manish Sharma, Heena Dixit

**Affiliations:** 1 Department of Dentistry, Government Medical College, Sheopur, Sheopur, IND; 2 Department of Oral Pathology, Swami Devi Dyal Hospital and Dental College, Barwala, IND; 3 Department of Periodontics, Government Dental College and Hospital, Srinagar, IND; 4 Department of Dentistry, Holyoke Health Center, Massachusetts, USA; 5 Department of Prosthodontics, Vishnu Dental College, Bhimavaram, IND; 6 Department of Oral and Maxillofacial Surgery, Dr. D. Y. Patil Dental College, Dr. D. Y. Patil Vidyapeeth (Deemed to be University), Pune, IND; 7 Department of Oral Pathology, Jawahar Medical Foundation's Annasaheb Chudaman Patil Dental College, Dhule, IND; 8 Department of Public Health, Commissionerate of Health and Family Welfare, Hyderabad, IND

**Keywords:** biomarker, corrosion, dental implants, saliva, titanium

## Abstract

Introduction: Dental implants are widely used for tooth replacement owing to their biocompatibility and long-term stability. Titanium released from implants into surrounding tissues and saliva may reflect corrosion and disease activity. This study aimed to evaluate salivary titanium ion levels in patients with healthy implants versus those with peri-implantitis one year after implant placement, and to assess their relationship with clinical parameters of peri-implant health.

Materials and methods: A prospective cohort study was conducted in 30 patients who received single titanium implants in the posterior region in the Department of Oral Pathology at Jawahar Medical Foundation's Annasaheb Chudaman Patil Memorial Dental College, Dhule, Maharashtra, India, from November 2022 to November 2023. Patients were divided into three groups (n = 10 each): no implant (control), healthy peri-implant mucosa, and peri-implantitis diagnosed using standardized clinical and radiographic criteria. Unstimulated whole saliva was collected at the 12-month follow-up using a standardized kit. The titanium concentration of the samples was analyzed using inductively coupled plasma mass spectrometry (ICP-MS). Clinical parameters, including probing depth and marginal bone loss, were recorded at six sites per implant. Data were compared between the groups using appropriate statistical tests.

Results: Salivary titanium levels were lowest in the no-implant group (3.3 ± 1.49 µg/L), significantly higher in patients with healthy implants (127 ± 13.32 µg/L), and highest in the peri-implantitis group (235.3 ± 17.94 µg/L). Probing depth and marginal bone loss followed a similar pattern, increasing from the control (0.21 ± 0.04 mm and 0.29 ± 0.07 mm) to healthy implant (3.14 ± 0.48 mm and 0.77 ± 0.13 mm) and peri-implantitis groups (4.87 ± 0.55 mm and 1.49 ± 0.23 mm). Statistical analysis confirmed significant differences in titanium levels, probing depth, and marginal bone loss across the groups (p = 0.001 for all). Post-hoc tests showed that peri-implantitis was associated with markedly greater values in all measured parameters compared with healthy implants and controls (p = 0.001 for all pairwise comparisons).

Conclusion: Salivary titanium ion concentration increased with the severity of peri-implant disease and correlated with the clinical indicators of inflammation and bone resorption. This non-invasive biomarker reflects implant surface degradation and may serve as an early diagnostic tool for peri-implantitis. Regular monitoring of salivary titanium could enhance risk assessment and support timely clinical intervention, thereby improving long-term implant outcomes.

## Introduction

Dental implants have revolutionized restorative dentistry by offering a durable and esthetically superior alternative to traditional prosthetics. Composed primarily of titanium (Ti) or its alloys, these implants exhibit excellent osseointegration owing to their biocompatibility and corrosion resistance in physiological environments [1]. However, despite these advantages, long-term success rates hover around 90-95%, with the prevalence of peri-implantitis and per-mucositis ranging from 19% to 65%, emerging as a primary cause of failure [2]. Peri-implantitis, characterized by progressive inflammation of the soft and hard tissues surrounding the implant, affects most implant patients within five to 10 years after post-placement, leading to bone loss, implant mobility, and eventual removal [3].

The pathogenesis of peri-implantitis involves a complex interplay between microbial biofilms, host immune responses, and biomaterial degradation [3]. Titanium, while inert, is not impervious to corrosion in the oral environment, where fluctuating pH, saliva composition, and mechanical stresses facilitate the release of Ti ions and particles [4]. These corrosion products can trigger local cytotoxicity, oxidative stress, and inflammatory cascades, exacerbating tissue breakdown [4]. Saliva, as a non-invasive diagnostic fluid, serves as an ideal matrix for detecting such biomarkers, reflecting systemic and local pathological changes with high sensitivity [5].

Emerging evidence suggests that salivary Ti ion levels may correlate with peri-implant health status [6]. In healthy mucosa, baseline Ti concentrations remain low (<1-2 µg/L), which is attributable to minimal wear and stable passivation layers [6,7]. There are controversial results regarding the levels of Ti in patients with peri-implantitis compared to those with healthy mucosa. Papi et al. [8] reported no significant differences in Ti levels between patients with peri-implantitis and those with healthy implants. Conversely, patients with peri-implantitis exhibit significantly elevated levels (up to five- to 10-fold), potentially due to accelerated corrosion from inflammatory mediators and bacterial by-products [9]. This differential expression positions salivary Ti ions as a promising chairside biomarker for early detection and monitoring, surpassing radiographic methods in accessibility and cost-effectiveness.

This study aimed to evaluate and correlate salivary Ti ion levels one year after implant placement in patients with healthy peri-implant mucosa versus those with peri-implantitis. The primary objective was to quantify and compare salivary Ti ion levels in individuals with healthy peri-implant mucosa and those with peri-implantitis at the 12-month follow-up. The secondary objective was to examine the association between salivary Ti concentrations and key clinical parameters, including probing depth and marginal bone loss, to evaluate how Ti release correlates with peri-implant disease severity. The null hypothesis stated that no significant difference existed in salivary Ti ion levels one year post-implantation between the groups.

## Materials and methods

This prospective cohort study was conducted in the Department of Oral Pathology at Jawahar Medical Foundation's Annasaheb Chudaman Patil Memorial Dental College, Dhule, Maharashtra, India, from November 2022 to November 2023. Ethical approval for this study was obtained from the Institutional Ethics Committee of the college, dated November 12, 2022 (EC/NEW/INST/2022/2959/SS115), in accordance with the Declaration of Helsinki and the Indian Council of Medical Research guidelines. All patients provided written informed consent prior to enrolment with detailed explanations of the study's purpose, procedures, potential risks (such as minimal discomfort during saliva collection or probing), and benefits (including free clinical monitoring).

The eligibility criteria were rigorously defined to ensure homogeneity and relevance. Inclusion criteria included adults aged 25-55 years who had received a single Ti dental implant (Adin Touche Deep Cone, Adin Dental Implant Systems Ltd., Petach Tikva, Israel) for partial edentulism in the posterior mandible or maxilla, with at least 12 months of functional loading post-placement, good general health (American Society of Anesthesiologists Class I or II), no systemic conditions predisposing to bone loss (such as uncontrolled diabetes, osteoporosis), and no history of head-neck radiation or bisphosphonate use.

Exclusion criteria included smoking (≥10 cigarettes/day), current periodontal therapy within six months, presence of multiple implants, or any peri-implant infection at baseline. Healthy peri-implant mucosa was defined as probing depth ≤4 mm, absence of bleeding or suppuration on probing, and no radiographic bone loss beyond 1 mm. Peri-implantitis was diagnosed according to the 2017 World Workshop criteria: probing depth ≥6 mm, bleeding/suppuration on probing, and confirmed radiographic bone loss ≥3 mm from the implant shoulder [10]. Group 1 (no-implant control) included systemically healthy adults who demonstrated clinically healthy periodontal status according to the 2017 World Workshop classification. Participants in this group had probing depths ≤3 mm, no clinical attachment loss, no radiographic bone loss, and bleeding on probing in ≤10% of sites. Individuals showing any signs of gingivitis, periodontitis, or a history of periodontal therapy within the previous six months were excluded to ensure a true healthy-periodontium control group. Participants who had taken systemic antibiotics within the last three months were excluded. Only the standard post-operative regimen (amoxicillin 500 mg TID for 5 days) following implant placement was allowed, and no additional antibiotic use occurred during the study period.

Sample size estimation

The sample size was determined using G*Power software (version 3.1.9.2, Heinrich Heine University, Düsseldorf, Germany) through an a priori power analysis for an F-test evaluating a fixed-effect omnibus with an effect size of 0.62, based on a prior work by Papi et al. [8] comparing Ti levels across three groups. The analysis yielded a total sample size of 30 (10 per group) to achieve 80% study power, with a 5% alpha error level. The general formula for the sample size calculation in a one-way analysis of variance (ANOVA) is as follows:

\begin{equation*} n = \frac{(Z_{1-\beta} + Z_{1-\alpha/2})^2 \cdot (k-1) \cdot \sigma^2}{k \cdot \delta^2} \end{equation*}

where:

n = sample size per group.

k = number of groups

σ = variance within groups.

δ= effect size between groups.

Z1-β= Z value for the statistical power at 80%

​​​​​​Z1-α= Z value for the significance level at 95%

Thirty selected patients were divided into three groups (n = 10 in each group): Group 1 served as a control, where no implants were placed; Group 2, healthy peri-implant mucosa; and Group 3, peri-implantitis. The study commenced with baseline implant placement in Groups 2 and 3 under sterile conditions in the department. Grade IV commercially pure titanium implants were surgically inserted following standard two-stage protocols, with primary stability confirmed via insertion torque (>35 Ncm) using an Adin surgical kit. Postoperative care included antibiotics (amoxicillin 500 mg three times daily for five days) and analgesics (ibuprofen 400 mg as needed). At the 12-month follow-up, clinical assessments were performed by a calibrated periodontist (intra-examiner reliability: κ=0.85). Probing depths and bleeding on probing were measured at six sites per implant using a plastic periodontal probe (PerioWise "Friendly Probe," Premier Dental Products Company, Pennsylvania, USA) to avoid Ti surface scratching. 

In the no-implant control group, periodontal probing was performed on the natural teeth adjacent to or corresponding to the anatomical sites where implants would typically be placed. Probing depths, bleeding on probing, and radiographic bone levels of these teeth were recorded to provide baseline periodontal health parameters for comparison with the implant groups. This ensured that measurements in the control group reflected healthy periodontal conditions rather than peri-implant tissues. Marginal bone loss was evaluated using standardized digital periapical radiographs taken with the paralleling technique to ensure reproducible angulation. The known implant length was used as a calibration reference in the imaging software, and bone levels were measured from the implant shoulder to the most coronal point of bone contact on both the mesial and distal surfaces. The average of these two measurements was recorded as the marginal bone level for each implant. All measurements were performed by a calibrated examiner to maintain consistency. The study baseline was considered after second-stage surgery, when the implants were uncovered and healing abutments were placed. Clinical evaluation and saliva collection were performed 12 months after second-stage surgery, ensuring that all implants had been in functional loading for one year. The period between first-stage surgery and second-stage surgery was not included in the follow-up duration, as the study specifically assessed peri-implant conditions after prosthetic loading.

The bleeding index was scored dichotomously (present/absent). Radiographic evaluation employed a digital periapical X-ray system (Orthophos SL 2D, Dentsply Sirona, North Carolina, USA) with a standardized paralleling technique (90 kVp, 7 mA, 0.1-second exposure), calibrated against a known implant length for accuracy. Marginal bone levels were quantified mesially and distally using image analysis software (Sidexis 4, Dentsply Sirona, North Carolina, USA), with an intra-observer variability of <0.5 mm.

Salivary Ti ion collection was performed immediately following clinical examinations to capture real-time peri-implant conditions. Unstimulated whole saliva was collected using the saliva collection kit (SuperSAL Universal, Oasis Diagnostics, Washington, USA), which employed an absorbent cylindrical pad placed along the buccal mucosa for one to three minutes until the volume adequacy indicator turned blue (>1 mL). Participants were instructed to abstain from eating, drinking, or oral hygiene for 90 minutes and rinse with distilled water 10 minutes before collection. Samples were centrifuged at 3,000 revolutions per minute (rpm) for 10 minutes (Eppendorf 5810R, Eppendorf AG, Germany) to remove debris, aliquoted into trace-metal-free tubes, and stored at -80°C until analysis. Ti concentrations were quantified via inductively coupled plasma mass spectrometry (iCAP RQ ICP-MS system, Thermo Fisher Scientific, Massachusetts, USA), calibrated with multi-element standards (0.1-100 µg/L titanium in 2% nitric acid as HNO3). Samples were diluted 1:10 in 2% HNO3 (trace-metal grade, Merck KGaA, Darmstadt, Germany) with internal standards (indium and bismuth) for drift correction. The detection limit was 0.01 µg/L, with quality control via certified reference material (Seronorm Trace Elements in Serum; Nycomed Pharma AS, Oslo, Norway), ensuring a relative standard deviation of <5 %. All procedures adhered to Good Laboratory Practice with a blinded analysis of group allocation.

Statistical analysis

Data were analyzed using IBM SPSS Statistics for Windows, version 26.0 (released 2018, IBM Corp., Armonk, NY). Categorical variables are expressed as frequencies and percentages. The normality of outcome data was assessed and confirmed using the Shapiro-Wilk test. Marginal bone loss, probing depth, and titanium levels were presented as means with standard deviations and 95% confidence intervals. Intergroup differences in these outcome variables were tested using one-way ANOVA followed by post-hoc Tukey's test for pairwise comparisons. Differences were considered statistically significant at a p-value < 0.05.

## Results

Table 1 shows the sex distribution across the three study groups. Among the 30 patients, 13 (43%) were female and 17 (57%) were male. Both sexes were evenly distributed between the groups. This balanced sex distribution facilitated an unbiased comparison of outcomes across groups.

**Table 1:** Sex distribution among the study groups. Values are presented as frequency (n) and percentage (%).

Groups	No implants		Healthy peri-implant mucosa		Peri-implantitis		Total	
Sex	n	%	n	%	n	%	n	%
Female	4	13%	4	13%	5	17%	13	43%
Male	6	20%	6	20%	5	17%	17	57%
Total	10	33%	10	33%	10	33%	30	100%

The descriptive analysis in Table 2 shows that the mean ages of the patients were comparable across the groups. Marginal bone loss was lowest in the no implant group (0.29 ± 0.07 mm) and progressively increased in the healthy implants (0.77 ± 0.13 mm) and peri-implantitis (1.49 ± 0.23 mm) groups, indicating bone resorption associated with implant presence and disease. Similarly, the probing depth was minimal in the no implant group (0.21 ± 0.04 mm), elevated in healthy implant patients (3.14 ± 0.48 mm), and highest in peri-implantitis patients (4.87 ± 0.55 mm), reflecting clinical inflammation in diseased implants. Ti levels in saliva markedly increased from no implant (3.3 ± 1.49 μg/L) to healthy implants (127 ± 13.32 μg/L) and peaked in peri-implantitis (235.3 ± 17.94 μg/L), suggesting Ti release is correlated with implant status and peri-implant disease severity. Overall, these findings highlight the progressively worsening clinical and biochemical parameters from no implant to peri-implantitis, supporting their use as markers of peri-implant health and disease.

**Table 2:** Descriptive statistics of study variables across the groups. Values are presented as mean ± standard deviation (SD) with 95% confidence intervals (CIs).

Variables	No implants		Healthy peri-implant mucosa		Peri-implantitis	
	Mean ± SD	95% CI for mean	Mean ± SD	95% CI for mean	Mean ± SD	95% CI for mean
Age (years)	30.5 ± 3.78	27.8 - 33.2	31.3 ± 4.50	28.08 - 34.52	28.5 ± 3.72	25.84 - 31.16
Marginal bone loss (mm)	0.29 ± 0.07	0.25 - 0.34	0.77 ± 0.13	0.68 - 0.86	1.49 ± 0.23	1.33 - 1.65
Probing depth (mm)	0.21 ± 0.04	0.18 - 0.24	3.14 ± 0.48	2.79 - 3.49	4.87 ± 0.55	4.48 - 5.26
Salivary titanium levels (µg/L)	3.3 ± 1.49	2.23 - 4.37	127 ± 13.32	117.47 - 136.53	235.3 ± 17.94	222.47 - 248.13

A one-way ANOVA test comparing the study groups revealed significant differences in marginal bone loss, probing depth, and salivary Ti levels. Therefore, the null hypothesis was rejected. The mean marginal bone loss was lowest in the control group without implants (0.29 ± 0.07 mm) and highest in the peri-implantitis group (1.49 ± 0.23 mm) (p = 0.001). Similarly, probing depth also followed the same pattern, with the lowest values in the control group without implants (0.21 ± 0.04 mm) and the highest in the peri-implantitis group (4.87 ± 0.55 mm) (p = 0.001). Salivary Ti levels showed marked elevation from 3.3 ± 1.49 μg/L in the no implant group to 127 ± 13.32 μg/L in the healthy implant and 235.3 ± 17.94 μg/L in the peri-implantitis group (p = 0.001), as shown in Table 3.

**Table 3:** Intergroup comparison among study groups using one-way analysis of variance (ANOVA). Values are presented as mean ± standard deviation (SD). *P < 0.05 denotes statistical significance (one-way ANOVA).

ANOVA test	No implants	Healthy peri-implant mucosa	Peri-implantitis	F value	p-value
Marginal bone loss (mm)	0.29 ± 0.07	0.77 ± 0.13	1.49 ± 0.23	149.39	0.001*
Probing depth (mm)	0.21 ± 0.04	3.14 ± 0.48	4.87 ± 0.55	310.81	0.001*
Salivary titanium levels (µg/L)	3.3 ± 1.49	127 ± 13.32	235.3 ± 17.94	806.00	0.001*

Post-hoc pairwise comparison revealed significant differences between groups across marginal bone loss, probing depth, and salivary Ti levels. Patients with peri-implantitis showed significantly higher marginal bone loss than healthy implants (t = 10.33; p = 0.001) and no implant groups (t = 9.15; p = 0.001). Similarly, probing depth was significantly greater in peri-implantitis implants than in healthy implants (t = 17.17; p = 0.001) and in no implant groups (t = 24.66; p = 0.001). Titanium levels also differed markedly, with peri-implantitis patients exhibiting higher levels than those with healthy implants (t = 6.84; p = 0.001) and without implants (t = 15.51; p = 0.001). These results confirm that peri-implantitis is associated with a progressive increase in marginal bone loss, probing depth, and salivary Ti ion release, supporting these measures as valuable indicators of implant health (Table 4).

**Table 4:** Pairwise comparisons between the study groups using post-hoc Tukey’s test. *P < 0.05 denotes statistical significance.

Post-hoc	Peri-implantitis: healthy peri-implant mucosa		Peri-implantitis: no implants		Healthy peri-implant mucosa: no implants	
Pairwise comparison	t value	p-value	t value	p-value	t value	p-value
Marginal bone loss	10.33	0.001*	9.15	0.001*	18.73	0.001*
Probing depth	17.17	0.001*	24.66	0.001*	40.12	0.001*
Salivary titanium levels	6.84	0.001*	15.51	0.001*	21.39	0.001*

## Discussion

The results of this study show a clear increase in salivary Ti ion levels in patients with no implants compared to those with healthy implants and the highest levels in patients with peri-implantitis. This pattern indicates that titanium release occurs even in clinically stable implants, however, is significantly greater in the presence of peri-implant disease.

Titanium release is mainly due to corrosion of the implant surface [11]. In healthy implants, mechanical forces from chewing cause micromovements that damage the protective oxide layer on titanium. Saliva contains electrolytes and has a varying pH, which promotes the gradual breakdown of this layer, leading to low-level ion release. Delgardo-Ruiz and Romanos [12] conducted a comprehensive analysis of the factors contributing to the liberation of Ti and Ti ions during implantation surgery, prosthetic implementation, and ongoing maintenance. They identified several risk factors, including mechanical influences, bacterial presence, saliva, micro-spatial configurations, and fluoride exposure. Similarly, Romanos et al. [13] noted that ejection of titanium particles may occur as a consequence of insertion, loading conditions, and maintenance practices.

In peri-implantitis, bacterial biofilms and inflammatory mediators worsen this process. Pathogenic bacteria produce acids that lower local pH and accelerate corrosion. At the same time, immune cells release cytokines and reactive oxygen, which further degrade the oxide layer [14]. This creates a cycle in which corrosion increases inflammation, which drives more corrosion. Safioti et al. [9] observed elevated levels of titanium within biofilms derived from tissues adjacent to peri-implantitis. The electrostatic properties and ionic bonding of titanium dioxide on the surface of implants may facilitate bacterial adhesion. The interplay between the metallic interface and oral environment could result in the liberation of degradation by-products from the implant into the peri-implant surroundings.

Noronha Oliveira et al. [15] reported that debris released from the degradation of dental implants is genotoxic and cytotoxic to the peri-implant tissues. Nanoparticle-coated dental implants have been found to promote osseointegration and reduce the risk of peri-implantitis [16]. The recent development of nano-engineered dental implants has significantly enhanced their corrosion resistance of dental implants [17].

Our findings are supported by those of several previous studies [18,19]. Previous studies have shown that dental implants increase salivary Ti levels [18,19]. Alrabeah et al. [19] reported the release of Ti ions from platform-switched and platform-matched dental implants, irrespective of the abutment size and configuration. Previous research has reported higher Ti levels in salivary samples from patients with dental implants as compared to the control group without dental implants [18].

The present study showed a progressive increase in marginal bone loss and probing depth from the no-implant group to healthy implants, and further to peri-implantitis. Marginal bone loss reflects peri-implant bone resorption driven by inflammation and mechanical stress, whereas probing depth indicates soft tissue inflammation and pocket formation around the implant. In peri-implantitis, bacterial plaques and host inflammatory responses trigger osteoclast activation, leading to greater bone loss and deeper pockets compared to healthy implants, where minimal changes occur due to stable osseointegration. These clinical parameters strongly correlated with elevated salivary Ti levels, suggesting that implant corrosion contributes to tissue breakdown and disease progression [9,11,12,15].

The strong association between salivary Ti levels, probing depth, and marginal bone loss suggests that Ti ions contribute to disease progression [9,15]. The released particles can trigger chronic inflammation by activating immune pathways, even when the bacterial load is reduced [11,12]. This may explain why some cases of peri-implantitis do not fully respond to mechanical cleaning alone. Monitoring salivary Ti levels could help to identify at-risk implants before severe marginal bone loss occurs.

Clinical implications

Salivary Ti ion measurement is non-invasive, quick, and can be performed during routine dental visits. Elevated levels may serve as early warning signs, prompting closer clinical evaluation or early intervention. This approach supports risk-based recall systems and may reduce the need for frequent radiography. It also opens the door for personalized maintenance protocols based on individual corrosion profiles.

Limitations

This study included a small sample size from a single center, which limits the generalizability of the results. The 12-month follow-up period may not reflect the long-term behavior of the Ti release. A microbial analysis was not performed; therefore, the role of specific bacteria remains unclear. In addition, the presence of other metal restorations in some patients could influence salivary Ti readings. Although the study excluded major confounders such as systemic diseases and smoking to improve internal validity, certain factors that may influence corrosion and titanium release were not controlled. Variations in individual oral hygiene practices, peri-implant plaque accumulation, implant surface characteristics, and prosthetic design could contribute to differences in titanium ion levels independent of disease status. These factors represent potential residual confounders that could not be fully eliminated despite standardized clinical and radiographic assessments. Future studies with more rigorous control or stratification of these variables are needed to better isolate their impact on titanium release and peri-implant tissue responses.

## Conclusions

This study demonstrated that salivary Ti ion levels increased progressively with worsening peri-implant conditions from healthy implants to peri-implantitis. Elevated Ti ion release results from surface corrosion, which is accelerated by mechanical loading, bacterial activity, and inflammatory mediators. These ions correlate with clinical signs of disease, including marginal bone loss and increased probing depth, indicating their role in peri-implant tissue breakdown. Salivary Ti ion measurement offers a non-invasive, practical biomarker for the early detection and monitoring of peri-implant health. These findings support its potential use in routine clinical practice to guide timely intervention and improve long-term implant success.
